# Steering mass transport with topologically protected sound

**DOI:** 10.1093/nsr/nwag121

**Published:** 2026-03-02

**Authors:** Jiuyang Lu, Zhengyou Liu

**Affiliations:** Key Laboratory of Artificial Micro- and Nanostructures of Ministry of Education and School of Physics and Technology, Wuhan University, China; Key Laboratory of Artificial Micro- and Nanostructures of Ministry of Education and School of Physics and Technology, Wuhan University, China; Institute for Advanced Studies, Wuhan University, China

Acoustic tweezers, which harness the radiation forces of sound waves to manipulate micro- and nanoparticles in a contact-free manner, have emerged as powerful tools in biology and medicine [1]. They enable precise handling of cells without the thermal damage often associated with optical tweezers. However, conventional standing-wave acoustic tweezers often lack flexibility, as the acoustic field typically covers the entire emission region, limiting the ability to define complex, arbitrary transport paths. While phononic crystals have offered some improvements, they generally suffer from poor robustness, where fabrication defects or sharp corners in the waveguide path can cause significant backscattering, disrupting the manipulation process.

Topological acoustics, inspired by topological concepts in condensed matter physics, offers a promising route to these limitations [2]. Topological interface states allow waves to propagate along specific boundaries with high transmission efficiency, immune to backscattering from sharp bends or structural imperfections [3]. While the propagation of topological acoustic waves has been widely studied, exploiting these robust fields for the practical, dynamic transport of matter has remained a significant challenge. Realizing such topological acoustic tweezers requires precise control over the interaction between the topological fields and the suspended particles to ensure stable trapping and movement in complex environments.

In a recent study published in *Science Advances*, a team led by Hairong Zheng at the Shenzhen Institutes of Advanced Technology and Xue-Feng Zhu at Huazhong University of Science and Technology demonstrated a robust topological acoustic tweezing platform [4]. By constructing a water-submerged phononic crystal composed of triangular steel pillars arranged to form a topological valley–Hall insulator (Fig. 1a), they established a valley interface capable of supporting robust interface waveguide states (Fig. 1b). Through precise phase modulation of the incident waves at 470 kHz, the researchers successfully generated localized standing-wave nodes and antinodes that could be continuously shifted (Fig. 1c and d). This setup allowed them to trap and transport particles along arbitrarily designed trajectories (Fig. 1e). Crucially, the mass transport was topologically protected, meaning it was unaffected by sharp Z-shaped corners or defects in the path. Such obstacles typically hinder transport in conventional acoustic waveguide devices. Furthermore, the system demonstrated intelligent capabilities, including path-selective transport and the realization of a topological mass circulator (Fig. 1f).

**Figure 1.**
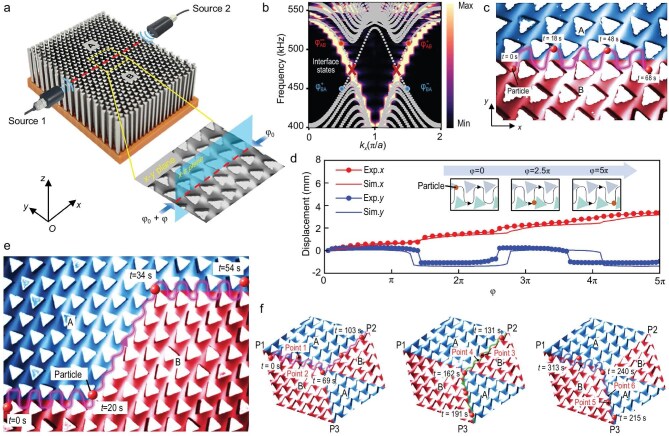
(a) Photograph of the experimental sample and schematic of the standing-wave excitation. (b) Dispersion of the interface waveguide state in the acoustic valley–Hall topological insulator. (c) Transport of polydimethylsiloxane spheres in a straight topological waveguide. (d) Particle displacement versus phase *φ*; insets display particle positions at increasing phase (left to right). (e) Robust mass transport in an acoustic topological waveguide with corner defects. (f) Mass transport in the topological acoustic circulator: from P1 to P2 (left), from P2 to P3 (middle), and from P3 to P1 (right). Reproduced from Huang *et al.* [4] with permission.

In summary, Zheng, Zhu and co-workers provide a compelling demonstration of how topological protection can be harnessed for robust mass transport. By integrating the robustness of topological physics with the manipulation capabilities of acoustic radiation forces, they have developed a system that maintains high efficiency, even in the presence of structural imperfections. This innovative approach significantly expands the toolkit for acoustofluidics [5], holding strong potential for advanced biomedical applications, such as precise cell sorting and targeted drug delivery.

## References

[1] Chen C, Gu Y, Philippe J et al. Nat Commun 2021; 12: 1118. 10.1038/s41467-021-21373-3 33602914 PMC7892888

[2] Xue H, Yang Y, Zhang B. Nat Rev Mater 2022; 7: 974–90. 10.1038/s41578-022-00465-6

[3] Lu J, Qiu C, Ye L et al. Nat Phys 2016; 13: 369–74. 10.1038/nphys3999

[4] Huang L, Xiang X, Li Z et al. Sci Adv 2026; 12: eadz4301. 10.1126/sciadv.adz4301 41477856 PMC12757033

[5] Zhao S, Tian Z, Shen C et al. Nat Mater 2025; 24: 707–15. 10.1038/s41563-025-02169-y 40119033 PMC12048345

